# Consultations and Consultants

**Published:** 1902-01-04

**Authors:** 


### The Hospital

A Journal of

### The Medical Sciences and Hospital Administration.

Vol. XXXI.—No. 797.] Edited by Sir Henry Burdett, K.C.B., and Solomon C. Smith, M.D., M.R.C.P. [January 4, 1902.

### CONSULTATIONS AND CONSULTANTS.

Few questions of greater difficulty present themselves in medical or surgical practice than those which arise out of a demand for consultations and consultants. Among the middle or upper middle classes of this country there is, as a rule, a distinct appreciation of the unwisdom of most endeavours to "swop horses when crossing a stream," and hence, in a case of illness of whatever seriousness, the family medical attendant, perhaps with the aid of a single consultant selected by himself, is usually left in absolute and unfettered control of the treatment, and, what is still more important, in a condition of full responsibility with regard to the case. When we come into the ranks of the "upper ten" a wholly new condition of affairs may have to be encountered. Relatives on both sides of the house, friends of the family, sometimes even exalted personages, are all urgent that the patient should have the benefit of the advice of Mr. A. or of Dr. B., and, where expense need not be considered, it seldom occurs to them that their well-meant counsel may really not be conducive to the recovery which it is intended to promote. "What did Lord X. die of?" said a friend of the deceased nobleman to one of the physicians who had stood at his bedside. "Of a committee without a chairman," was the reply. A lady of rank who died a few years ago, and who for a long period had been a recognised invalid, was in the habit of seeing many different advisers from day to day during her sojourn in town, and, prior to her departure for the country, was accustomed to have a consultation of them all. On one occasion, we have heard, no less than thirteen medical carriages were in waiting at her door while this final consultation was being held; and it is not difficult to conjecture what amount of benefit to the patient was likely to be derived from it.

There are, of course, many cases, or at all events some, which present unquestionable, and possibly for the time insuperable, difficulties of diagnosis; and in all these there is at least the possibility that any one possessing adequate knowledge, who comes to the case unbiassed by preconceptions, may observe some clue to the truth which has been overlooked by others. In the great majority of instances, no such doubts exist; and the state of the patient at once declares itself to skilled examination. It demands, let us say, certain definite medical treatment, and proper regulation of diet, temperature, and other surroundings; all of them matters which are receiving careful attention from the family doctor and from the first consultant who has been called in. In a surgical case the chief necessity may be for an operation; and the only questions admitting of dispute may be such as might arise during its course, and require prompt determination on the spur of the moment. In such circumstances, what is an additional consultant to do? If he be strong, wise, and conscientious, he may be able to say that he has no suggestion to make. In the majority of instances, however, strength, wisdom, and conscience are matters of degree; more especially, perhaps, among the medical favourites of the general or of the fashionable public. An additional consultant culled from among these will be likely to feel that something is expected of him. He has to do something for his fee and to justify his presence. He will suggest an almond poultice softened with rose-water, or, perhaps, the substitution of strophanthus for digitalis.

What are the men in possession to do? It is their plain duty to defend the patient against any change of treatment which in their opinions would not be to his advantage, and they may therefore feel it incumbent upon them to repudiate the strophanthus. The poultice can do no harm, and they submit for the sake of peace, more or less holding a candle to the devil, and expecting to obtain a similar concession when they are themselves placed in similar circumstances. The patient recovers, and it is all the third doctor and his poultice, with the natural consequence that he is pushed by his particular patronesses even more strenuously than before. In the matter now under consideration, we are much inclined to think that the profession generally would be entirely justified in keeping a more firm control over the multiplication of consultants than has of late years been customary, and in asserting the right of those who have conducted a case of illness through its earlier and often more doubtful stages, to remain in undisturbed control of it until its close. The "safety" which Solomon attributed to a multitude of counsellors has its source, if at all, in the certainty it may afford that nothing is left unnoticed ; but it does not extend to the elevation of "a committee without a chairman" to the position of a machinery calculated to bring about the restoration of health to the sick.

